# Bee venom exhibits anti-cancer effects on tongue carcinoma cells by arresting cell cycle, inducing apoptosis, and suppressing cell migration

**DOI:** 10.1590/1678-7757-2025-0188

**Published:** 2025-08-11

**Authors:** Enas SABRY, Hagar M ZAYED, Ola M EZZATT, Iman FATHY, Hebatt-Allah S ELSAYEH, Nashwa EL-KHAZRAGY, Suzan SeifAllah IBRAHIM

**Affiliations:** 1 Ain Shams University Faculty of Dentistry Department of Oral Medicine,Periodontology and Oral Diagnosis Cairo Egypt Ain Shams University, Faculty of Dentistry, Department of Oral Medicine, Periodontology and Oral Diagnosis, Cairo, Egypt.; 2 Ain Shams University Faculty of Dentistry Department of Oral Biology Cairo Egypt Ain Shams University, Faculty of Dentistry, Department of Oral Biology, Cairo, Egypt.; 3 Ain Shams University Faculty of Dentistry Central Lab of Stem Cells and Biomaterial Applied Research Cairo Egypt Ain Shams University, Faculty of Dentistry, Central Lab of Stem Cells and Biomaterial Applied Research (CLSBAR), Cairo, Egypt.; 4 Plant Protection Research Institute Agricultural Research Center Ministry of Agriculture Giza Egypt Beekeeping Research Department, Plant Protection Research Institute, Agricultural Research Center, Ministry of Agriculture, Giza, Egypt.; 5 Ain Shams University Faculty of Medicine Department of Clinical Pathology/Hematology Cairo Egypt Ain Shams University, Faculty of Medicine, Department of Clinical Pathology/Hematology, Cairo, Egypt.; 6 Nahda University Faculty of Oral and Dental Medicine Beni Suef Egypt Nahda University, Faculty of Oral and Dental Medicine, Beni Suef, Egypt.

**Keywords:** Apitoxin, Apis mellifera, Cancer therapy, Honeybee, HNO-97 cell line

## Abstract

**Objective:**

Tongue squamous cell carcinoma (TSCC) is an aggressive oral cancer with notable treatment resistance. This *in vitro* study investigated anti-cancer effects of honey bee venom (BV)—a mixture of bioactive compounds—on the human TSCC cell line.

**Methodology:**

The cytotoxicity of serial BV concentrations (0.01–100 µg/mL) was tested on the cultured human TSCC cell line (HNO-97) to determine the half-maximal inhibitory concentration (IC_50_) value. Group I (BV) included cells treated with IC_50_ of BV, and Group II (control) received no treatment; both were incubated for 48 hours. The apoptotic effect of BV was evaluated using the Annexin V assay and the BAX and BCL-2 gene expression. The BV effect on cell viability, proliferation, and division was evaluated by cell cycle assay. Additionally, Transwell migration assays were performed to demonstrate the potential impact of BV on cell migration.

**Results:**

BV showed dose-dependent cytotoxicity and anti-proliferative activity on HNO-97 cells (IC_50_: 12.96 μg/mL). The treated group exhibited cell cycle arrest, reduced cell migration, significantly decreased BCL-2 gene expression (p=0.001), and increased BAX gene expression (p=0.03) compared to the untreated group.

**Conclusion:**

BV demonstrated anti-cancer activity on human TSCC by inducing apoptosis and inhibiting cell migration. These findings warrant further preclinical investigations to evaluate BV as an alternative for current tongue carcinoma therapies.

## Graphical abstract

**Figure:**
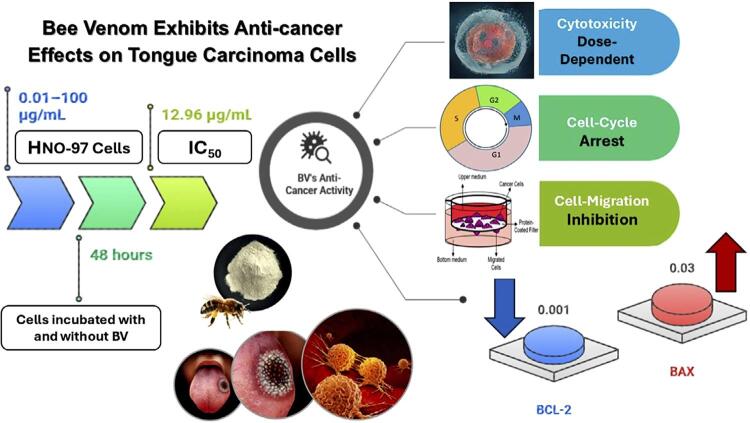

## Introduction

Oral squamous cell carcinomas represent 90% of all malignancies in the head and neck region. Interestingly, tongue cancer is a global health burden amongst other oral squamous cell carcinomas, with increasing incidences among young populations in developing countries.^1^ Like other cancers, tongue squamous cell carcinoma (TSCC) is a cell cycle disease, in which cancer tissues experience abnormal proliferation due to disruptions in the cell cycle regulatory system. Furthermore, cell apoptosis is a process of programmed cell death, crucial in cancer progression.^2^ The regulation of intrinsic apoptosis pathways involves a family of gene-controlled proteins that can promote or inhibit apoptosis.^3^

Current management strategies for oral cancer include surgical excision, chemotherapy, radiotherapy, and combined therapy, depending on tumor stage and the patient’s medical condition. Chemotherapeutic agents such as methotrexate, 5-fluorouracil, and cisplatin are commonly used antimetabolites in TSCC treatment but are associated with severe adverse reactions, toxic side effects, and drug resistance. Furthermore, these agents may not effectively target less-active cancer cells, influencing the overall recurrence and prognosis.^4^ Thus, TSCC management remains challenging for clinicians and biomedical researchers due to its aggressive nature, treatment resistance, and complications or side effects associated with conventional chemotherapy.^5^

Natural compounds derived from bacteria, plants, or other animal species have been critical in advancing cancer treatments due to their extensive chemical structure and diverse biological activities.^6^ Bees produce various bioactive components, including honey, propolis, royal jelly, bee pollen, beeswax, and bee venom (BV), also known as apitoxin.^7^ *Apis mellifera* is the most common honeybee species in the Middle East and produces BV, which contains phospholipase A2, melittin, polyamines, catecholamines, and histamine—a mixture of active proteins, enzymes, antibacterial agents, antioxidants, and anti-cancer compounds. Melittin (MEL), representing approximately 50% of BV, has been reported to have prominent anti-cancer effects.^8^

Apitherapy, which involves using bees or their products for therapeutic or preventive purposes, has a long history in traditional medicine; BV has been used as a pain reliever and to treat inflammatory diseases such as multiple sclerosis and rheumatoid arthritis. It has also been applied to treat various illnesses, including spinal cord injuries, Parkinson’s disease, and Alzheimer’s disease.^9^

A recent review summarizing anti-cancer effects of BV in numerous human cancer cell lines and animal models showed that BV effectively induces apoptosis, inhibits angiogenesis, arrests the cell cycle, and reduces cancer cell invasion and metastasis.^10^ BV also enhances the cytotoxic effects of cisplatin in several head and neck squamous cell carcinomas,^11^ including TSCC.^12^This growing body of evidence suggests that BV could be a potential alternative to conventional chemotherapeutic medications.

Given the limited *in vitro* and preclinical studies exploring the sole effect of BV on human tongue squamous cell carcinoma, this study is designed to investigate the anti-cancer effect of BV in terms of cytotoxic, anti-proliferative, apoptotic, and anti-migratory properties on the human TSCC cell line.

## Methodology

The study was approved by the Research Ethics Committee of the Faculty of Dentistry at Ain Shams University (FDASU-Rec ID IM04230). Consent was not required because human samples were not used in this study.

### Bee venom and vehicle control solution preparation

BV collection and preparation were conducted at the Department of Bee Research, Plant Protection Research Institute, Agriculture Research Centre, Giza, Egypt**.** Colonies of the *Apis mellifera L.* honeybee, of the first Carniolon hybrid strain, were used. BV was collected every three days from March to November 2023, between 4:00 and 6:00 p.m., using an electric BV collector device. Under stimulation by a low-voltage current (18–22 V) (Electric shock device, VC-Starter kit; IGK Electronics Ltd., Varna, Bulgaria), the bees secreted a thick yellowish, suspension with a characteristic odor resembling honey. The powdered BV was then lyophilized (freeze-dried) to produce light gray to grayish-yellow powder, which was stored at -20°C.^13^

A 100µg/mL stock solution was prepared by dissolving 0.1 gram of lyophilized BV powder in 1 mL of distilled water. The solution was then filtered using a 0.4μm paper filter and stored in a black bottle at 4°C until used.^14^ Before the cell cytotoxicity test, 10 serial concentrations from the stock solution were prepared as follows: 0.01, 0.03, 0.1, 0.3, 1.0, 3.0, 10.0, 30.0, 70.0, and 100 µg/mL, using 0.1% dimethylsulfoxide (DMSO)(Gibco, Thermosientific, Germany) as the diluent. A 0.1% DMSO solution was used as vehicle control.^11^

### Cell line and culturing

TSCC cell line, HNO-97 (GmbH, Eppelheim, Germany) was obtained via Nawah Scientific’s Cell Line services at the Department of Cell Culture, Cairo, Egypt. Cells were cultured in T- 25 mL flasks (Thermo Fisher Scientific, Massachusetts, USA) containing 15 mL of Dulbecco’s Modified Eagle Medium (DMEM), composed of 4.5 g/L glucose, 4 mM L-glutamine, 1.5 g/L NaHCO3, and 1.0 mM sodium pyruvate (Gibco). The medium was supplemented with 10% fetal bovine serum (FBS; Gibco), and 1% of penicillin G sodium (10.000 UI; Gibco), streptomycin (10 mg; Gibco), and amphotericin B (25 μg; Gibco). Cells were incubated at 37°C with 5% CO_2_ using a Midi 40 small-capacity CO2 incubator (Thermo Fisher Scientific, USA).

When cells reached 80% confluence, they were separated using 0.25% trypsin-EDTA solution prepared in calcium- and magnesium-free phosphate-buffered saline (Thermo Fisher Scientific) according to manufacturer instructions (Supplementary File). Cells were sub-cultured at a plating density of 1x10^6^ cells per flask, and those from the fourth passage were used for further analysis.

### Cell cytotoxicity assay

The effect of different BV concentrations on HNO-97 cell viability was analyzed using the 4.5-dimethylthiazol-2-yl-2.5 diphenyltetrazolium bromide (MTT) assay (Vybrant^®^ MTT Cell Proliferation Assay Kit, cat. #: M6494, Thermo Fisher, Germany). The test was performed following the standard protocol.^15^ Briefly, 8×10^3^ passaged cells in 200 μL culture media were seeded per well in a 96-well culture plate (Thermo Fisher Scientific). An equal volume of phosphate-buffered saline (PBS) was added to control wells. Wells were grouped in triplicates (three replicates/concentration in addition to the control group), then incubated for 24 h at 37°C to enable overnight cell attachment. A 20 µL MTT solution (1mg/mL)was added to each well and incubated at 37°C and 5% CO_2_ for four hours. Finally, 100 μL of sodium dodecyl sulfate with hydrochloric acid (SDS-HCL) was added to each well after discarding the MTT solution.

The half-maximal stimulatory concentration (IC_50_) was calculated for each group based on concentration-response curves of cellular metabolic activity using the GraphPad prism software (Prism 9, version 9.1.0-221). Cell viability was determined by measuring the optical density (OD) at a 570 nm wavelength using a spectrophotometer (ELx 800; Bio-Tek Instruments Inc., Winooski, VT, USA). The viability percentage for each concentration was calculated using the formula: mean OD of test concentration/ mean OD of negative control solution x100.^16^

### Grouping and application of treatment

In this *in vitro* study, cultured HNO-97 cells were seeded in a 6-well plate (Thermo Fisher Scientific) at a concentration of approximately 1×10^6^ cells/2 mL of complete media. Wells were divided into two groups: Group I (BV), which received treatment with IC_50_ of BV, and Group II (control), which received a 0.1% DMSO solution as a vehicle control with no treatment. The whole plate was incubated for 48 hours at 37°C with 5% CO_2_.

### Morphological examination of cultured cells

Cells from both groups were examined under an inverted microscope with a mounted Vega digital camera (Labomed, USA) to observe morphological changes in response to treatment.

### Cell cycle assay

BV-treated HNO-97 cells were harvested 48 hours post-treatment. Following trypsinization, cells were fixed with cold methanol and stained with Vybrant^®^ DyeCycle™ Violet (Invitrogen, cat. #: V35003. ThermoFisher, USA). Stained cells were incubated at 37°C for 30 minutes, protected from light until analysis. DNA content across different cell cycles was examined using flow cytometry. Using 405 nm excitation at 440 nm emission, the percentage of cells in G0, G1, S, and G2M phases was analyzed to determine the distribution of viable, necrotic, early apoptotic, and late apoptotic cells using a Navios EX flow cytometer and software (Beckman Coulter, USA) .^17^

### Apoptosis assessment

#### Detection of apoptotic cells by annexin V/propidium Iodide (PI) staining

Apoptotic cell were detected using the Annexin V/PI Apoptosis Kit (Alexa Fluor^®^ 488 annexin V/Dead Cell Apoptosis Kit, Invitrogen, cat. #: V13241, ThermoFisher, USA). The assay utilizes recombinant annexin V conjugated to green-fluorescent Alexa Fluor^®^ 488 dye, along with red-fluorescent propidium iodide (PI), a nucleic acid-binding dye that is impermeant to live and apoptotic cells but stains dead cells. After staining, cell populations were categorized using a flow cytometer (Beckman Coulter) using 488 nm excitation and emissions at 530 nm and 575 nm. Early apoptotic cells showed green fluorescence, late apoptotic cells showed green and red fluorescence, dead or necrotic cells showed red fluorescence only, and live cells showed little or no fluorescence.^18^

#### Measuring the expression of apoptotic genes by Real-Time PCR

Total RNA was extracted from HNO-97 cells in both groups (1x10^6^ cells in 350µL of the lysis buffer) using the Tissue Ruptor II (Qiagen, Hilden, Germany) and the RNeasy Mini kit (Qiagen). The extracted RNA was then reverse transcribed using the QuantiTect Reverse Transcription Kit (Qiagen), following the manufacturer’s instructions.^19^

Expression levels of BAX (BCL2 Associated X, Apoptosis Regulator) and BCL2 (B-Cell Leukemia/Lymphoma 2) genes were assessed using quantitative real-time polymerase chain reaction (qRT-PCR) with the following reagents:

QuantiTect primer assays: Hs_BAX_1_SG (cat. #: 249900, ID: QT00031192), Hs_BCL2_1_SG (cat. #: 249900, ID: QT0025011), and Hs_ACTB_1_SG (β-actin; cat. #: 249900, ID: QT00095431) as the housekeeper gene; and SYBR Green PCR Kit (QuantiTect, cat. #: 204141, (Qiagen).

RT-PCR primers were as follows:

**BCL-2**:

Forward: 5′-CTTTGAGTTCGGTGGGGTC A-3′

Reverse: 5′-GGGCCGTACAGTTCCACAAA-3′

**BAX**:

Forward: 5′-GGCCCTTTTGCTTCAGGGTT-3′

Reverse: 5′-GGAAAAAGACCTCTCGGGGG-3′

**β-actin:**

Forward: 5′-TTCCAGCCTTCCTTCTTG-3′

Reverse: 5′-GGAGCCAGAGCAGTAATC-3′

Cycling and amplification were performed according to the manufacturer’s protocol. All samples were analyzed in triplicate by measuring threshold cycle (Ct) values in the exponential phase of amplification using the 5-plex Rotor Gene PCR Analyzer (Qiagen). The relative expression level for the target gene was calculated based on the 2^− Ct^ method (ΔCt = Ct [gene of interest] – Ct [housekeeper gene]), (2^− Ct^ = ΔCt [experimental sample] – ΔCt [control sample]). All values were normalized to β-actin (ACTB) levels and displayed as fold changes.^20^

## Cell migration assay

The Transwell migration assay evaluated the HNO-97 cell migratory behavior following BV treatment. The test was performed using an 8 mm caliber Boyden Chamber (Thermo Fisher Scientific, Germany). Briefly, 600 μL of IC_50_ was added to the cells plated on top of the filter membrane in a Transwell insert and incubated at 37°C and 5% CO2 for 48 hours. Adherent cells migrated and attached to the other side of the membrane, while non-adherent cells that migrated dropped into the media in the lower chamber.

At the end of the incubation period, the Transwell insert was removed and placed into 70% ethanol for 10 minutes to enable cell fixation. Then, 600μL of 0.2% crystal violet was added to a well of 24-well plate, and the membrane was positioned in the solution for staining, incubated at room temperature for 5–10 minutes. The membrane was subsequently examined under an inverted microscope with a mounted Vega digital camera (Labomed, USA). The number of migrated cells was determined by counting cells in multiple fields of view, calculating the average number of cells that migrated through the membrane toward the chemo-attractant and attached to its underside.^21^ Cell migration percentage was calculated as: *Percentage of Migration = (Migrated Cell Area/Total Membrane Area) × 100.*

## Statistical analysis

Statistical analysis was performed using the Statistical Package for Social Sciences (SPSS) version 20 (SPSS, Inc., Chicago, IL, USA). Assays were repeated independently in triplicate. For the MTT assay, intergroup comparisons were made using ANOVA followed by Tukey’s post hoc test. Independent t-tests were used for intergroup comparisons. Flow cytometry data, expressed as percentages, were compared between groups using the chi-square test. Results were presented as mean ± standard deviation (SD) and confidence intervals. P-values ≤0.05 were considered statistically significant.

## Results

### The cytotoxic effect of BV on HNO-97 cells

**Figure 1:**
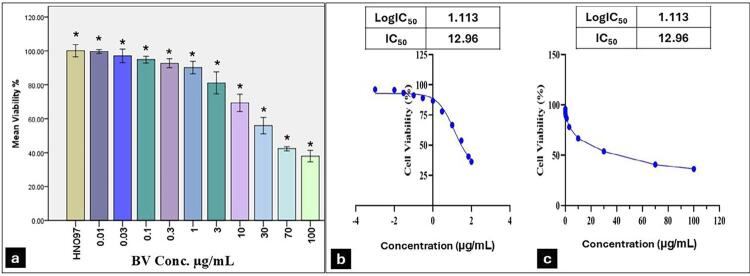
BV effect on HNO-97-cells viability and its calculated IC50. (a) The bar chart shows a reduction in mean and standard deviation values for cell viability (%) with an increased BV concentration of 0.01-100μg/mL. Data is expressed as the mean±S.D; * indicates statistically significant differences from the control (p≤0.05). (b) The linear regression curve illustrates the log dose. (c) BV concentration (μg/mL) versus the normalized response in HNO-97 cells after treatment with serial concentrations in DMEM for 48 hours, showing IC50 of 12.96 μg/mL

**Figure 2:**
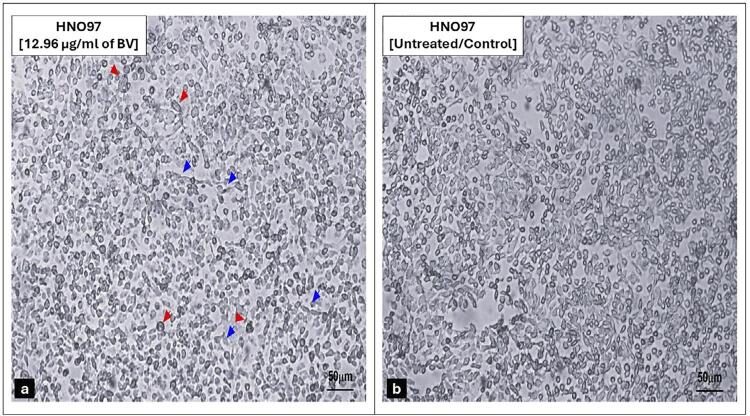
Morphological examination of cultured human tongue squamous cell carcinoma (HNO-97) in the two study groups. (a) BV (12.96 μg/mL)-treated HNO-97 cells appear rounded, shrunken, and occasionally irregular in shape and floating (red arrow), while a few spindle-shaped cells remain adherent to the culture flask surface (blue arrow). (b) un-treated HNO-97 cells (fourth passage) showed adherent colonies of the confluent viable spindle-shaped cell monolayer. Magnification (20x). Scale bar=50μm.

The mean percentage of viable cells in the treated group statistically decreased (p=0.0001) as drug concentrations increased from 0.01µg/mL to 100 µg/mL, indicating a dose-dependent cytotoxicity effect (Figure 1a). The BV concentration needed to reduce HNO-97 cell viability by 50% (IC_50_) was estimated at 12.96 μg/mL (Figure 1b). Morphological examination of BV-treated cells for 48 hours showed clear signs of cell death, shrinkage, and detachment (Figure 2a) compared to uniformly attached cell colonies of untreated cells (Figure 2b).

### The effect of BV on the HNO-97 cell cycle

**Figure 3:**
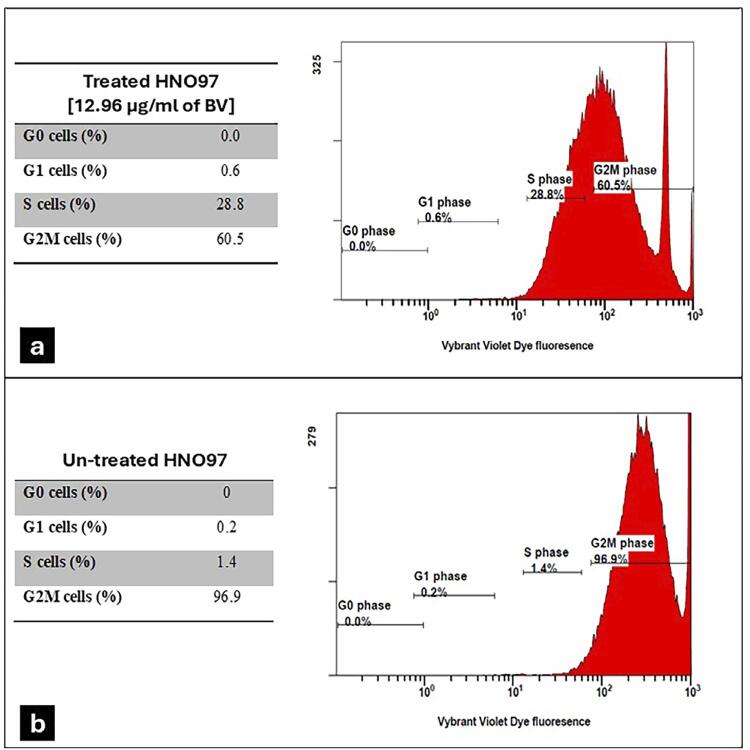
Histograms of flow cytometry DNA content distribution in a cell cycle analysis assay. (a) HNO-97 cells treated with 12.96µg/mL of BV showed that 0% were in G0, 0.6% were in G1, 28.8% were in S stage, and 60.5% were G2/M cells. (b) Untreated HNO-97 cells showed a lower percentage of cells in the S stage (1.4%) and a higher percentage in the G2/M phase (96.9%).

The cell cycle assay of BV-treated cells showed percentages of 0% in G0, 0.6% in G1, 28.8% in S, and 60.5% in G2/M phases. In comparison, the untreated group showed cell percentages of 0%, 0.2%, 1.4%, and 96.9% in these respective cell cycle stages. Notably, BV treatment significantly reduced cell percentage in the G2/M phase along and increased the percentage in the S phase. These differences between groups were statistically significant (p=0.0001) (Figures 3a, b).

### Evaluation of BV effect on HNO-97 cell apoptosis

**Figure 4:**
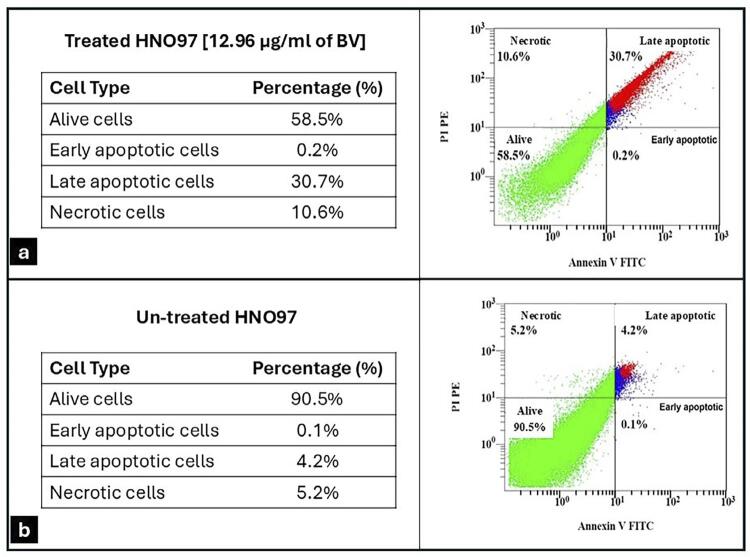
Flow cytometry histograms from apoptosis assay using Annexin V/PI staining LL: Viable cells showed little or no fluorescence (Annexin V −/ PI −), LR: early apoptotic cells showed green fluorescence (Annexin V +/ PI −), UL: dead/necrotic cells showed red fluorescence (Annexin V −/ PI +) and UR: late apoptotic showed green and red fluorescence (Annexin V +/ PI +). (a) HNO-97 cells treated with 12.96 µg/mL of BV showed that (30.7%) were late apoptotic cells, (10.6%) were necrotic, and (58.5%) were alive cells. (b) Untreated HNO-97 cells showed a lower percentage of late apoptotic cells (4.2%) and necrotic cells (5.2%) and a higher percentage of alive cells (90.5%).

**Figure 5:**
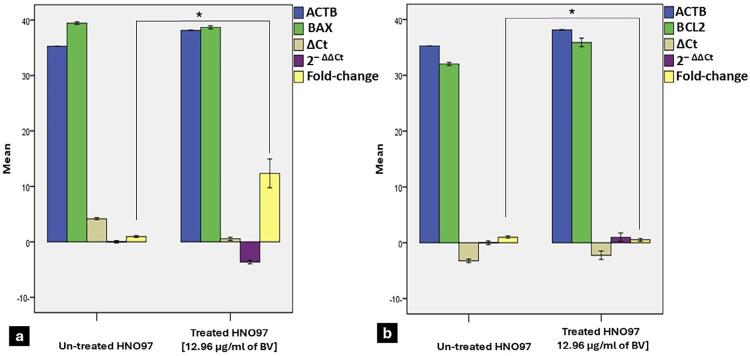
Bar charts of gene expression levels of BAX and BCL2 Assay (ACTB: β-actin, Ct: cycle threshold, ΔCt: difference of expression, 2− ΔΔCt: difference in ΔCt between test and control). (a) BAX gene expression was significantly higher in HNO-97 cells treated with 12.96µg/mL of BV when compared to untreated cells (p=0.003). (b) BV-treated HNO-97 cells demonstrated significantly lower BCL2 expression compared to untreated cells (p=0.003). Data is expressed as mean±S.D; * indicates statistically significant differences from the control (p≤0.05).

The apoptosis assay showed a significantly higher percentage of apoptotic and necrotic cells at in BV-treated HNO-97 cells compared to untreated cells (p=0.0001) (Figures 4a, 4b). Analysis of apoptosis-related gene expression showed that BAX expression in treated HNO-97 cells increased significantly, reaching approximately 12.35-folds compared to 1-fold in untreated cells (p=0.003) (Figure 5a). Conversely, BCL-2 expression was significantly lower in the treated group (approximately 0.51-fold) compared to the control group (1-fold) (p=0.003) (Figure 5b).

### BV effect on the migration ability of HNO-97 cells

**Figure 6:**
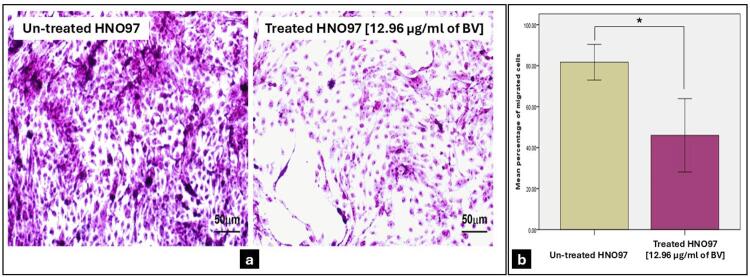
Transwell Migration Assay. (a) Photomicrographs illustrate the difference in migrated untreated HNO-97 cells and cells after treatment with 12.96µg/mL of BV for 48 hours. Magnification (40x). Scale bar=50μm (b) The bar chart shows that BV-treated cells had a significant reduction in the mean percentage of migrating cells compared to untreated cells (p=0.002). Data is expressed as the mean±S.D; * indicates statistically significant differences from the control (p≤0.05).

The Transwell migration assay revealed a significant reduction (p=0.002) in the mean percentage of migrating cells in the BV-treated group, which reached a 46% reduction compared to untreated cells, which reached 81.67% (Figures 6a, b).

## Discussion

To the authors’ knowledge, unlike prior studies that investigated the synergistic effects of BV with cisplatin,^12^ this study investigated BV effects as a sole treatment. This provides a baseline for future therapeutic techniques targeting human TSCC (HNO-97), a cancer known for its resistance to conventional drugs such as cisplatin).^4^ Our findings offer new insights into the anti-cancer effect of BV, demonstrating its ability to arrest the cell cycle, induce apoptosis, and suppress cell migration in tongue carcinoma cells. This *in vitro* design was also essential toward clinical translation by characterizing BV as a sole chemotherapeutic agent for tongue carcinoma and determining the concentration required to kill 50% of the cancer cell population within 48 hours (IC_50_). Previous studies have reported that BV can selectively induce anastasis in normal cells, while promoting irreversible death in cancer cells via cytosine-triggered alterations.^22,23^ Thus, a normal cell line was not included as a control group in this study.

BV showed dose-dependent cytotoxicity and anti-proliferative activity on HNO-97 cells, and an IC_50_ value of 12.96 μg/mL. In a study conducted on another tongue carcinoma cell line (SCC-25), IC_50_ values were 1.56 μg/mL for BV-treated cells.^12^Another study on four different types of head and neck squamous cell carcinoma (UMSCC12, UMSCC29, UMSCC38, and UMSCC47) documented IC_50_ of BV 54.809 µg/mL, 61.287 µg/mL, 71.328 µg/mL, and 61.045 µg/mL.^11^ BV also inhibited the growth of human colon cancer cells HCT116, and the IC_50_ value was ٨.٢ μg/mL.^24^

All previous studies demonstrated the dose-dependent cytotoxic effect of BV, which can be attributed to its composition of many substances with anti-proliferative properties. Among these melittin is key, as it preferentially binding to tumor cells over healthy cells, inducing lysis of the tumor cell membrane and inhibiting proliferation.^25^ However, IC_50_ values varied across different cancer cell lines. These variations were constantly observed and could be attributed to initial cell seeding density, BV collection method^26^, and the proliferation potential of cell lines. These factors were also demonstrated in a systematic review examining the effect of a single treatment on different oral squamous cell carcinoma cell lines.^27^

Flow cytometric analysis of the cell cycle confirmed that BV induced cell cycle arrest at the G2/M phase and enhanced the percentage of S-phase cells. Similar results were observed in other head and neck squamous cell carcinoma cell lines,^11^ as well as in other cancer cells such as breast, hepatocellular, and colorectal cancer cell lines.^28^

The observed distribution pattern suggested that most untreated cells were actively cycling and accumulated in the G2/M phase under the experimental conditions. In contrast, BV-treated cells showed cell cycle arrest predominantly in the S and G2/M phases, suggesting a potential inhibitory effect on DNA replication and mitotic progression, ultimately leading to apoptosis—a sign of increased cytotoxicity against cancerous cells.^29^ Although the distribution of untreated cells differs from the typical quiescent profile reported in other studies (which is usually predominantly in the G0/G1 phase), our findings were consistent across replicates and may reflect specific characteristics of the cell line or culture conditions used. The significant increase of apoptotic and necrotic cells within BV-treated cells compared to untreated cells further confirmed the inhibitory effect of BV on tongue cancer cell growth.

BV treatment significantly increased the expression of the BAX gene (proapoptotic) while decreasing the expression of BCL-2 (antiapoptotic) compared to untreated cells. The BAX to BCL-2 ratio is an indicator of susceptibility to apoptosis; thus, the alteration in the expression of these genes promote the release of apoptogenic proteins that trigger apoptosis in the cancer cells.^30^ These findings are consistent with previous studies on similar cell lines,^11,12^ and may be attributed to melittin activity, the major component of BV, to discharge endonuclease G and apoptosis-inducing factors from mitochondria—key elements in caspase-independent apoptotic pathways.^31,32^ While the Annexin V/PI assay and BAX/BCL2 expression analysis support complementary information on apoptosis initiation, priming, and execution, further investigations are necessary clearly differentiate between caspase-independent and caspase-dependent apoptotic pathways by means of caspase-3 cleavage detection.

In this study, BV-treated cells revealed a significant decrease in the percentage of migrating cells compared to untreated cells. Cell viability remained at 58.5% following treatment, suggesting that the reduced migration was not primarily due to cytotoxic effects. However, complementary assays—such as wound-healing and live-cell imaging—are recommended in future studies to more accurately distinguish migratory effects from viability-related effects. These results indicated BV ability to prevent malignant cell migration, which is crucial in cancer progression and metastasis. Similarly, melittin inhibited the migration of epidermal growth factor-induced invasion and migration of non-small cell lung cancer cells.^33^ BV-treated cervical cancer cells exhibited time-dependent reductions in motility compared to untreated cells.^34^

In a former study by Jo, et al., the treatment with BV and melittin significantly induced dose-dependent apoptosis in ovarian cancer cells, achieving rates of approximately 55–65% and 60–80%, respectively.^35^ Similarly, Mahmoodzadeh, et al.^36^ (2015) investigated the impact of melittin on gastric cancer cells and observed enhanced apoptosis and necrosis following treatment. Zhang, et al.^37^ (2017) further reported that melittin effectively inhibited the migration and invasion of non-small cell lung cancer cells stimulated by epidermal growth factor. More recently, Pandey, et al.^38^ (2023) comprehensively reviewed studies on anti-cancer melittin properties derived from BV across various human cancer models. The review highlighted the ability of melittin to induce apoptosis, arrest the cell cycle, modulate oncogenic signaling pathways, inhibit metastasis, and enhance chemo/radiotherapy sensitivity.

Generally, analysis of BV collected and prepared similarly to the methodology of this study showed that its main active components are melittin (52.1%), phospholipase A2 (11.9%), and apamin (2.3%).^39^ The apoptosis/BCL-2 effects align most closely with the known mechanisms of melittin,^32^ while reducing cell migration could involve the anti-inflammatory action of phospholipase A2.^8^ These findings suggest that the BV anti-cancer effects are driven by the synergistic action of its collective components rather than the isolated effect of each constituent, thereby enhancing its therapeutic potential.

## Conclusions

This study presented compelling evidence that BV holds significant potential as a natural anti-cancer agent, exerting its effects by inducing apoptosis, halting cell cycle progression, and preventing cell migration in tongue carcinoma cells. This suggests that BV could be effective in controlling and treating this cancer. However, additional research is required to examine the impact of BV on an experimental *in vivo* model of tongue carcinoma, aiming to identify the optimal therapeutic dose necessary for clinical translation.
